# The effect of caffeine, nap opportunity and their combination on biomarkers of muscle damage and antioxidant defence during repeated sprint exercise

**DOI:** 10.5114/biolsport.2023.112088

**Published:** 2021-12-31

**Authors:** Mohamed Romdhani, Nizar Souissi, Ismail Dergaa, Imen Moussa-Chamari, Yassine Chaabouni, Kacem Mahdouani, Olfa Abene, Tarak Driss, Karim Chamari, Omar Hammouda

**Affiliations:** 1 High Institute of Sport and Physical Education of Sfax, Sfax University, Sfax, Tunisia; 2 Physical activity, Sport and health, UR18JS01, National Observatory of Sports, Tunis, Tunisia; 3 PHCC, Primary Health Care Corporation, Doha, Qatar; 4 College of Education, Physical Education Department, Qatar University; 5 Department of biochemistry, CHU Ibn Jazzar, Kairouan, Tunisia; 6 Laboratory of Analysis, Treatment and Valorization of Pollutants of the Environment and Products (LATVEP) Faculty of pharmacy, University of Monastir, Monastir, Tunisia; 7 Regional center of sport medicine, Kairouan, Tunisia; 8 Interdisciplinary Laboratory in Neurosciences, Physiology and Psychology: Physical Activity, Health and Learning (LINP2), UFR STAPS, UPL, Paris Nanterre University, Nanterre, France; 9 ASPETAR, Qatar Orthopedic and Sports Medicine Hospital, Doha, Qatar; 10 Research Laboratory, Molecular Bases of Human Pathology, LR19ES13, Faculty of Medicine, University of Sfax, Sfax, Tunisia

**Keywords:** Psychostimulant, Daytime sleep, Ergogenic aid, Inflammation, Oxidative stress, High-intensity exercise

## Abstract

To investigate the effect of 20 min nap opportunity (N20), 5 mg · kg^-1^ of caffeine (CAF) and their combination (CAF+N20) on the biochemical response (energetic biomarkers, biomarkers of muscle damage and enzymatic antioxidants) to the running-based anaerobic sprint test. Fourteen highly trained male athletes completed in a double-blind, counterbalanced and randomized order four test sessions: no nap with placebo (PLA), N20, CAF and CAF+N20. Compared to PLA, all treatments enhanced maximum and mean powers. Minimum power was higher [(mean difference) 58.6 (95% confidence interval = 1.31–116) Watts] after CAF and [102 (29.9–175) Watts] after CAF+N20 compared to N20. Also, plasma glucose was higher after CAF [0.81 (0.18–1.45) mmol · l^-1^] and CAF+N20 [1.03 (0.39–1.64) mmol · l^-1^] compared to N20. However, plasma lactate was higher [1.64 (0.23–3.03) mmol · l^-1^] only after N20 compared to pre-exercise, suggesting a higher anaerobic glycolysis during N20 compared to PLA, CAF and CAF+N20. Caffeine ingestion increased post-exercise creatine kinase with [54.3 (16.7–91.1) IU · l^-1^] or without napping [58.9 (21.3–96.5) IU · l^-1^] compared to PLA. However, superoxide dismutase was higher after napping with [339 (123–554) U · gHB^-1^] or without caffeine [410 (195–625) U · gHB^-1^] compared to PLA. Probably because of the higher aerobic glycolysis contribution in energy synthesis, caffeine ingestion resulted in better repeated sprint performance during CAF and CAF+N20 sessions compared to N20 and PLA. Caffeine ingestion resulted in higher muscle damage, and the short nap enhanced antioxidant defence with or without caffeine ingestion.

## INTRODUCTION

Napping has received increased attention in the last few years with a generally observed ergogenic effect on physical and cognitive performances [1, 2], whether after partial sleep deprivation (PSD) [3–6] or after a normal sleep night (NSN) [7, 8]. Further, the post-lunch dip (PLD) has been described as the perfect time to nap because of the higher sleep propensity and the shorter sleep latency [9, 10]. Indeed, napping at 14h00 and 15h00 produced better physical performance at 17h00 compared to napping at 13h00 [8]. One limitation to daytime napping is sleep inertia, which refers to the feeling of disorientation immediately upon awakening [11]. Caffeine ingestion could be an effective countermeasure to sleep inertia that could exist after daytime napping [12]. Thus, caffeine could be ingested shortly before a daytime nap to maximize the gain obtained from the nap. A recent study reported that, on a relatively small sample size, a short nap enhanced repeated sprint performance more than caffeine, and the combination of CAF+N20 enhanced repeated sprint performance more than napping alone or caffeine ingestion alone after PSD [3]. However, a recent study showed that such a combination was not any better than each treatment alone on reaction time in highly trained athletes [13]. Indeed, the effect of caffeine and napping and their combination on repeated sprint performances requires further investigations with larger sample size.

The ergogenic effect of caffeine ingestion on athletic performance depends on several aspects (e.g., the caffeine dose, the duration and quality of prior sleep, and the task itself) [13, 14]. Several mechanisms have been suggested to be behind the ergogenic effect of caffeine. For instance, counteracting adenosine action in the central nervous system is regarded as the primary mechanism of caffeine’s action [15, 16]. Adenosine is a sleep-promoting substance that increases with wake duration and during physical exercise [17, 18], and caffeine antagonizes its actions [19]. For physical exercise, increasing catecholamine plasma concentration would increase glycogenolysis, glucose uptake and circulatory adjustments to the exercise [15]. Also, caffeine ingestion enhances forced respiratory volume in asthmatic and healthy subjects and during exercise [20]. One limitation to caffeine ingestion before exercise is the enhanced muscle damage [3, 21, 22], with or without performance enhancement [3]. During high-intensity exercise, the sarcolemma could be damaged due to metabolic and mechanical factors, resulting in muscle enzymes leaking into the circulation [23]. Indeed, phagocytic cells and cytokines are recruited to repair the damaged muscles. Also, exercise increases oxygen consumption and consequently free radical production, which could create a state of imbalance in favour of free radicals, known as oxidative stress. Therefore, muscle damage and oxidative stress are secondary to the increased metabolic and mechanical output during the exercise. However, it has been reported that napping enhances antioxidant defence against the exercise-induced oxidative stress after PSD [4] and after NSN [7].

The current study aimed to investigate the effect of 20 min nap opportunity (N20), a moderate dose (i.e., 5 mg · kg^-1^) of caffeine (CAF) and their combination (CAF+N20) on the biochemical response (energetic biomarkers, biomarkers of muscle damage and enzymatic antioxidant) to repeated sprint exercise. Based on the existing literature, it was expected that (i) napping will enhance repeated sprint performance more than caffeine ingestion, (ii) the combination of a moderate dose of caffeine and a short nap will result in better performance than each alone, (iii) caffeine ingestion will increase the exercise-induced muscle damage, and (iv) napping will enhance antioxidant defence.

## MATERIALS AND METHODS

### Ethics approval

The present study was approved by the University of Manouba Institutional Review Board (P-SC N° 009/15) and was conducted according to the ethical guidelines of the Declaration of Helsinki (64^th^ World Medical Association General Assembly, Fortaleza, Brazil, October 2013).

### Participants

Twenty volunteers met the inclusion criteria and only fourteen completed the study. They were all male, highly trained judokas and competing at the international level (20.43 ± 1.22 years, 174.86 ± 8.77 cm, 73.07 ± 11.72 kg, BMI = 23.85 ± 3.12 kg·m^-2^). Only caffeine naïve (< 80 mg · day^-1^), non-habitual nappers, non-smokers and drug-free athletes were recruited. All of them were moderate or intermediate chronotype (scored between 31 and 69) according to the Horne and Östberg [24] morningness/eveningness questionnaire. During the month preceding the experiment, sleep diaries were collected and only participants who scored ≤ 5 according to the Pittsburgh Sleep Quality Index [25] were included.

### Experimental design

The current study is part of a bigger project investigating the effect of caffeine consumption and napping of different durations on biochemical response to repeated sprint performance during the postlunch dip. Part of the current study’s results, comparing the effects of 20 and 90 min nap opportunity, was published elsewhere [7].

Before starting the main experiment, participants underwent two habitation sessions. In them, they were familiarized with the experimenters, laboratory, used material/devices, the sleeping room, tests, and questionnaires.

**FIG. 1:**
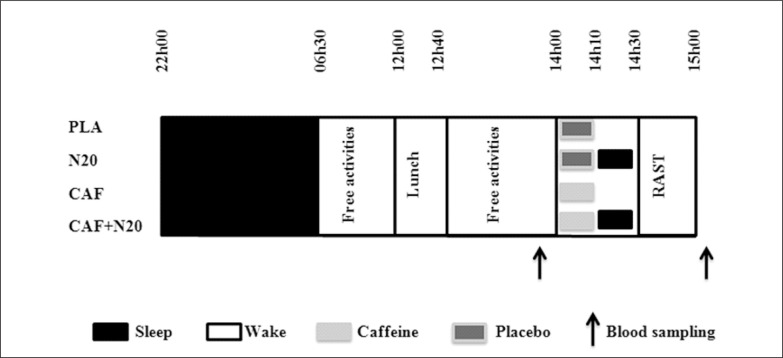
Simplified experimental protocol. Note: PLA: placebo, N20: 20 min nap opportunity with placebo, CAF: 5mg · kg^-1^ of caffeine session, CAF+N20: 5 mg · kg^-1^ of caffeine + 20 min nap session. RAST: running-based anaerobic sprint test, h: hour. All times are expressed in local time (GMT+1h).

Four test sessions were held at least one week apart (i.e., for washout) in a double-blind counterbalanced and randomized order (no nap with placebo ingestion (PLA), 20 min nap opportunity with placebo ingestion (N20), intake of 5 mg · kg^-1^ of caffeine without napping (CAF) and intake of 5 mg·kg^-1^ of caffeine prior to N20 (CAF+N20, Fig. 1). During each session, participants came to the laboratory at ~20h00, and consumed a standardized dinner at ~20h30. After that, they were free to watch television, play video games or surf on the internet, until 22h00 when they went to bed (all lights and devices off). Participants were aroused at 06h30 (~08h30 of time in bed), which corresponds to their daily routine. After a qualitatively and quantitatively standardized breakfast at 07h00, they stayed awake until 12h00 doing the same passive activities as in the previous evening (no food allowed, drinking water *ad libitum)*. At 12h00, they took a standardized iso-caloric lunch and stayed lying on a comfortable armchair. At 14h00, participants with the nap condition entered a room that was conducive to sleep (comfortably cool, fully dark and quiet). At this time, volunteers ingested 5 mg · kg^-1^ of pure powdered caffeine for CAF+N20 or cellulose and starch-based placebo for N20 [12]. The desired amount of caffeine (± 0.1 mg) was measured using a specific electronic weighing machine (Shimadzu, Shimadzu Corporation, Kyoto, Japan) and put into capsules to match the placebo in weight, colour and smell. After this, they wore earplugs and eye masks and got into bed; they were allowed a 20 min nap opportunity (from 14h10 to 14h30). Participants with the no-nap conditions ingested CAF or PLA at the same time (i.e., 14h00). Constant supervision was enabled by an infrared camera connected, in real time, to the experimenter’s computer in order to monitor the participants’ activities. When the nap period ended, participants were awakened by an alarm placed next to the bed. Upon awakening, participants subjectively rated their sleep on a 100 mm analogue scale; ranging from 0 “no sleep at all” to 100 “deep, uninterrupted sleep”. After the awakening, 30 min was allowed to overcome any sleep inertia [3–5, 7]. The period between 13h00 and 15h00 in the no-nap conditions (i.e., PLA and CAF) was spent watching a neutral documentary lying on a comfortable armchair. Finally, the running-based anaerobic sprint test (RAST) started at 15h00, followed by post-exercise (i.e., 5 min of passive recovery) blood sampling.

The laboratory conditions were fixed during all experimental days; temperature ~25°C (± 1.8°C), humidity ~35% (± 3.2%), and luminosity (i) ~200 lux during tests, and (ii) < 5 lux during sleep.

### Protocols

#### Running-based anaerobic sprint test (RAST)

RAST (six 35 m straight-line sprints with 10 s recovery in between for the turnaround) was performed [26].

#### Rating of perceived exertion (RPE)

The CR-10 psycho-physiological scale given score assessed the exertion which the athlete experience during the exercise [27].

#### Blood sampling and analysis

Blood samples were collected and analysed as previously described by Romdhani et al. [3, 4, 7, 28]. Table 1 presents all the methods used in the sample analysis.

**TABLE 1:** Different methods used in blood samples analysis.

Parameters	Method
Lactate [La]	Lactate oxidase peroxidase method (intra and inter-assay coefficient of variation (CV) were: 0.9% and 1.9%, respectively)
Glucose (GLC)	Glucose hexokinase method (intra and inter-assay CV were: 0.9% and 1.3%, respectively)
Aspartate Aminotransferase (AST)	Kinetic method at 340 nm
Creatine Kinase (CK)	Kinetic method at 340 nm
Urea (URE)	Kinetic enzymatic method (intra and inter-assay CV were: 0.3% and 5.6%, respectively).
Glutathione Peroxidase (GPx)	Spectrophotometric method based on Paglia and Valentine (1967) method (with Ransel RS. 505 kit, from Randox; Randox Laboratories Ltd. Crumlin, County Antrim, UK). The intra and inter assays CV were: 7.3 and 4.8%, respectively.
Superoxide Dismutase (SOD)	SOD activity in erythrocytes was measured by the rate of inhibition of 2-(4-iodophenyl)-3-(4-nitrophenol)-5-phenyltetrazolium chloride (INT) reduction. The kit used in this method was from Randox Lab (Ransod, RX MONZA). 0.5 ml of whole blood was centrifuged and then separated from the plasma. Erythrocytes were washed four times with 3 ml of 0.9% NaCl solution and centrifuged after each wash. 2.0 ml with cold redistilled water was added to the resulting erythrocytes, mixed and left to stand at +4°C for 15 minutes. A 25 fold dilution of lysate was then added. The intra and inter assays CV were: 5.9 and 4.6% respectively.

#### Statistical analyses

The statistical tests were processed using GraphPad Prism 6 (GraphPad Software, San Diego, CA, USA). The Shapiro-Wilk test revealed that data were normally distributed. Hence, parametric tests were used. For RAST and [La], a one-way analysis of variance (ANOVA) with repeated measures (4 treatments) was used. For biochemical parameters, a two-way repeated measures ANOVA was used (before/after the nap/rest × 4 treatments). To assess the ANOVA practical significance, eta-squared (η^2^) was calculated. Once the ANOVA indicated a significant main effect or interaction effect, the Bonferroni post-hoc test was used to check differences. Furthermore, the effect size (*d*) was calculated according to Cohen [29] to determine the amplitude of the difference in pairwise comparisons. The magnitude of *d* was classified as small (*d* < 0.5), moderate (0.5 ≤ *d* < 0.8) or large (*d* ≥ 0.8). Further, mean difference (*MD*) and the 95% confidence interval (95% CI) are provided for pairwise comparison. All values within the text, figures, and tables are reported as mean ± standard deviation (SD). The level of significance was set at p < 0.05.

## RESULTS

### Participants

The sample size was a priori calculated using the G*power software [30], based on literature evidence [3, 4] and following the procedure suggested by Beck [31]. The G*power software indicated a minimal required sample size of 12 participants. It was expected that not all the participants would finish the protocol appropriately [4]. Therefore, twenty volunteers were screened, from whom only fourteen participants’ data are included in the statistical analysis. With the abovementioned considerations, the actual power of the study design was 0.96.

### Sleep quality during the nap

Participants rated their sleep quality during the nap immediately upon awakening. The one-way ANOVA showed a significant main effect of napping on sleep quality (F_(3,13)_ = 59.4, p < 0.001, η^2^ = 0.82). Subjective sleep quality was higher after N20 (p < 0.001, *d* = 1.12, *MD* = 1.43, 95% CI = 0.42 to 2.43) compared to CAF+N20.

### Running-based anaerobic sprint test and lactate

**FIG. 2:**
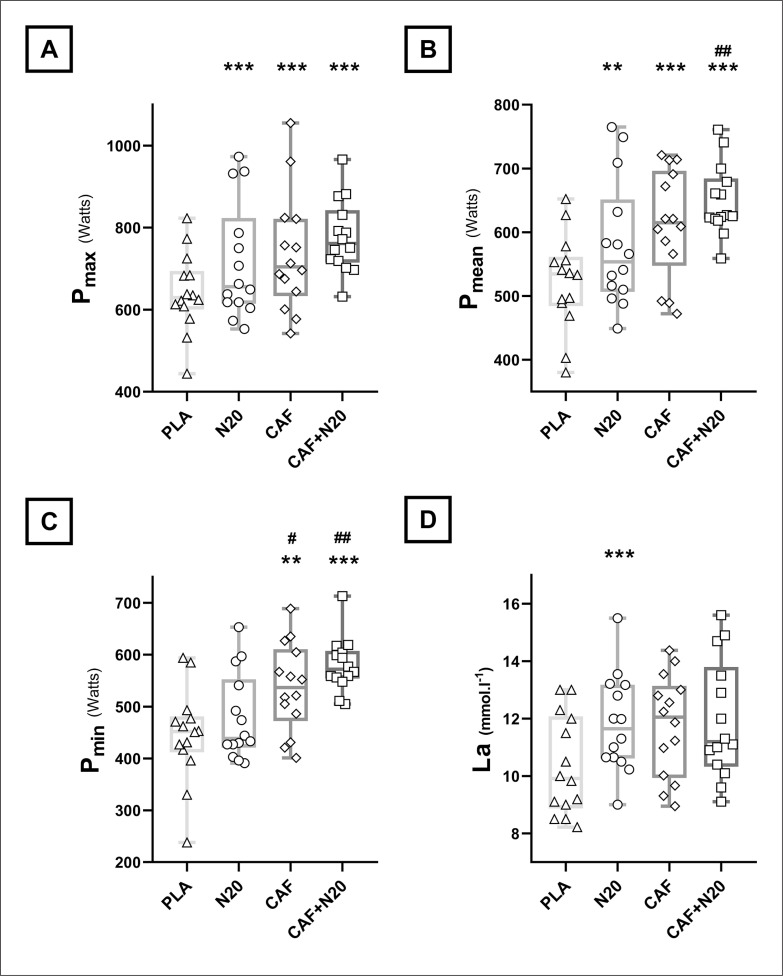
Group means and standard deviation with individual values of maximum (P_max_; A), mean (P_mean_; B) and minimum (P_min_; C) powers and plasma lactate concentrations [La; D] after different protocol sessions. Note: PLA: placebo, N20: 20 min nap opportunity with placebo ingestion, CAF: 5 mg · kg^-1^ of caffeine and CAF+N20: 5 mg · kg^-1^ of caffeine + 20 min nap opportunity. ^*^, ^**^ and ^***^ indicate a significant difference in comparison with PLA session at p < 0.05, p < 0.01 and p < 0.001, respectively; ^#^, ^##^ and ^###^ indicate a significant difference in comparison with N20 values at p < 0.05, p < 0.01 and p < 0.001, respectively.

Compared to PLA, all treatments enhanced P_max_ and P_mean_ (Fig. 2A, 2B & Table 2a). The increase of P_mean_ was higher after CAF+N20 compared to N20 (p = 0.04, *d* = 0.87, *MD* = -69.9 Watts, 95% CI = -138 to -2.2 Watts). P_min_ increased after CAF and CAF+N20 compared to PLA (Fig. 2C & Table 2a), but not after N20. Indeed, P_min_ was higher after CAF (p = 0.043, *d* = 0.71, *MD* = -58.6 Watts, 95% CI = -116 to -1.31 Watts) and CAF+N20 (p = 0.004, *d* = 1.49, *MD* = -102 Watts, 95% CI = -175 to -29.9 Watts) compared to N20. RPE and FI were unchanged across different protocol sessions.

**TABLE 2a:** RAST and [La]’s ANOVA output and pairwise comparison.

	ANOVA (One-way)			PLA vs. N20				PLA vs. CAF				PLA vs. CAF+N20			
	F_(3,13)_	p	η^2^	p	*d*	*MD*	95% CI	p	*d*	*MD*	95% CI	p	*d*	*MD*	95% CI
**P_max_** (W)	11.3	< 0.001	0.46	< 0.01	0.59	-72.9	-126 to -19.4	< 0.01	0.77	-94.5	-162 to -27.4	< 0.001	1.47	-135	-206 to -65.1
**P_mean_** (W)	17.1	< 0.001	0.56	< 0.05	0.66	-57.7	-112 to -3.1	< 0.001	1.12	-90.2	-137 to -43.1	< 0.001	1.94	-128	-187 to -68.7
**P_min_** (W)	15.5	< 0.001	0.54	NS	0.37	-33.6	-92.3 to 25.1	< 0.01	1.05	-92.2	-162 to -22.7	< 0.001	1.85	-136	-207 to -65.7
**[La]** (mmol · l^-1^)	3.74	< 0.05	0.22	< 0.05	0.89	-1.64	-3.03 to -0.23	NS	0.9	-1.57	-3.17 to 0.04	NS	0.88	-1.74	4.14 to 0.64

### Energetic markers

**FIG. 3:**
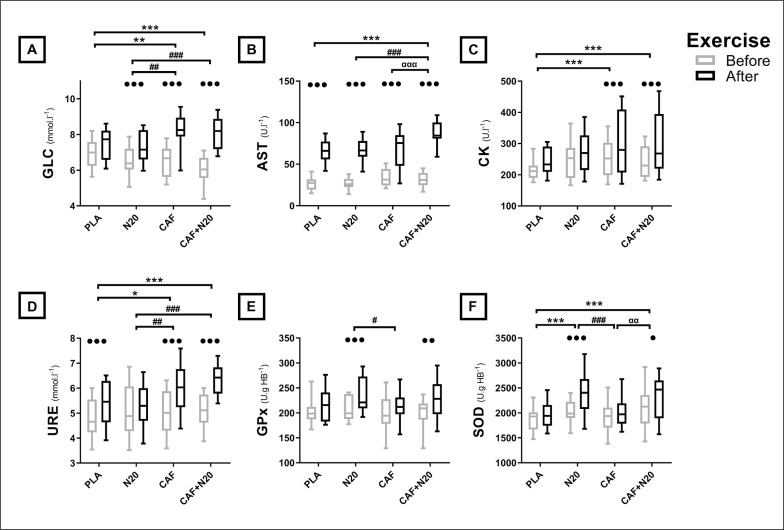
Group means and standard deviation of GLC: plasma glucose (A) AST; aspartate amino transferase (B), CK; creatine-kinase (C), URE; urea (D), GPx; glutathione peroxidase (E) and SOD; superoxide dismutase (F) before and after the exercise during different protocol sessions. Note: PLA: placebo, N20: 20 min nap opportunity with placebo ingestion, CAF: 5 mg · kg^-1^ of caffeine, CAF+N20: 5mg · kg^-1^ of caffeine + 20 min nap opportunity. ^●^, ^●●^ and ^●●●^ Significant effect of exercise at p < 0.05, p < 0.01 and p < 0.001 respectively, ^*^, ^**^ and ^***^ Significant difference in comparison with PLA at p < 0.05, p < 0.01 and p < 0.001 respectively, ^#^, ^##^ and ^###^ Significant difference in comparison with N20 at p < 0.05, p < 0.01 and p < 0.001 respectively, ^α^, ^αα^ and ^ααα^ Significant difference in comparison with CAF at p < 0.05, p < 0.01 and p < 0.001 respectively.

[La] increased significantly only after N20 compared to PLA (Fig. 2D & Table 2a). Compared to pre-exercise levels, post-exercise plasma glucose (GLC) increased after all treatments except PLA. This increase was more marked after CAF (p < 0.01, *d* = 0.92, *MD* = -0.81 mmol·l^-1^, 95% CI = -1.45 to -0.18 mmol·l^-1^) and CAF+N20 (p < 0.001, *d* = 1.03, *MD* = -1.03 mmol·l^-1^, 95% CI = -1.64 to -0.39 mmol·l^-1^) compared to N20 (Fig. 3A & Table 2b).

**TABLE 2b:** Biochemical parameters’ ANOVA output and pairwise comparison.

ANOVA (Two-way interaction)				PLA vs. N20				PLA vs. CAF				PLA vs. CAF+N20			
	F(3,39)	p	η2	p	*d*	*MD*	95% CI	p	*d*	*MD*	95% CI	p	*d*	*MD*	95% CI
**GLC** (mmol · l^-1^)	9.99	< 0.01	0.49	NS	0.06	0.06	-0.56 to 0.69	< 0.01	0.86	-0.75	-1.39 to -0.12	< 0.001	0.96	-0.93	-1.61to -0.27
**AST** (UI · l^-1^)	4.42	< 0.01	0.28	NS	0.07	-0.92	-13.1 to 11.3	NS	0.08	-1.64	-13.8 to 10.5	< 0.001	1.52	-21.2	-33.4 to -9.04
**CK** (UI · l^-1^)	1.03	NS	0.14	NS	0.36	-33.9	-71.5 to 3.67	< 0.001	0.8	-58.9	-96.5 to -21.3	< 0.01	0.76	-54.3	-91.1 to -16.7
**URE** (mmol · l^-1^)	4	< 0.05	0.29	NS	0.06	0.06	-0.56 to 0.69	< 0.05	0.74	-0.68	-1.28 to -0.09	< 0.001	1.31	-0.99	-1.58to -0.39
**GPx** (U · gHB^-1^)	1.26	NS	0.13	NS	0.53	-18.4	-39.1 to 2.39	NS	0.19	6.14	-14.6 to 26.9	NS	0.34	-12.1	-32.8 to 8.68
**SOD** (U · gHB^-1^)	2.84	< 0.05	0.24	< 0.001	1.16	-410	-625 to -195	NS	0.78	-305	-567 to -41	< 0.001	0.91	-339	-554 to -123

Note: RAST; the Running-based Anaerobic Sprint test, [La]: Plasma Lactate, Pmax: maximum power during the RAST, Pmean; mean power during the RAST, Pmin; minimum power during the RAST, W; watts, ANOVA; analysis of variance, N20; 20 min nap opportunity, CAF; 5 mg · kg^-1^ of caffeine, CAF+N20; 5 mg · kg^-1^ of caffeine + 20 min nap opportunity, F; Fisher’s F, p; probability, η^2^; Eta-squared, *d*; Cohen’s effect size, *MD*; Mean difference, 95% CI; 95% confidence Interval, NS; Non-significant, GLC; Plasma Glucose, AST; Aspartate Aminotransferase, CK; Creatine Kinase, URE; Urea, GPx; Glutathione Peroxidase and SOD; Superoxide Dismutase.

### Biomarkers of muscle damage

Post-exercise aspartate aminotransferase (AST) increased after all conditions (Fig. 3B & Table 2b). However, this increase was larger after CAF+N20 compared to N20 (p < 0.001, *d* = 1.47, *MD* = -20.3 U · l^-1^, 95% CI = -32.5 to -8.1 U · l^-1^) and CAF (p < 0.001, *d* = 1.03, *MD* = -19.6 U · l^-1^, 95% CI = -31.8 to -7.39 U · l^-1^). Post-exercise creatine kinase (CK) and urea (URE) increased whenever participants ingested caffeine (i.e., after CAF and CAF+N20) compared to PLA (Fig. 3C, 3D & Table 2b). Moreover, URE was higher after CAF (p < 0.01, *d* = 0.81, *MD* = -0.74 mmol·l^-1^, 95% CI = -1.34 to -0.15 mmol · l^-1^) and CAF+N20 (p < 0.001, *d* = 1.42, *MD* = -1.05 mmol·l^-1^, 95% CI = -1.64 to -0.45 mmol·l^-1^) compared to N20.

### Enzymatic antioxidants

Post-exercise glutathione peroxidase (GPx) and superoxide dismutase (SOD) increased only when participants napped, whether after placebo or caffeine ingestion (Fig. 3E, 3F and Table 2b). The increase of GPx and SOD was higher after N20 (p < 0.05, *d* = 0.75, *MD* = 24.5 U · gHB^-1^, 95% CI = 3.75 to 45.2 U · gHB^-1^; p < 0.001, *d* = 1.02, *MD* = 369 U · gHB^-1^, 95% CI = 154 to 584 U · gHB^-1^, respectively) compared to CAF. Further, post-exercise SOD was higher after CAF+N20 compared to CAF (p < 0.01, *d* = 0.77, *MD* = -297 U · gHB^-1^, 95% CI = -512 to -81 U · gHB^-1^).

## DISCUSSION

The main finding is that all interventions enhanced maximum (P_max_) and mean (P_mean_) powers during the RAST and only caffeine ingestion enhanced minimum power (P_min_), with or without napping. Also, CAF+N20 enhanced repeated sprint performances more than CAF alone or N20 alone. Furthermore, napping enhanced antioxidant defence with placebo or caffeine ingestion, and caffeine increased the exercise-induced muscle damage with or without napping.

A short nap enhanced P_max_ and P_mean_ but not P_min_ during repeated sprint, similarly to earlier reports after PSD [3, 4] and NSN [7]. Likely, the ergogenic effect of the short nap dissipated as the exercise became longer, which favours the short nap when the subsequent effort is brief [4]. However, a moderate dose of caffeine enhanced RAST performance more than the short nap, contrarily to our first hypothesis. In an earlier study, it was reported that N20 enhanced P_max_ but CAF had no effect on repeated sprint performance compared to PLA [3]. The fact that athletes were sleep deprived in the former study could explain this conflicting result. Indeed, the same dose of caffeine (i.e., 5 mg · kg^-1^) did not result in any repeated sprint enhancement in sleep deprived athletes, but it enhanced repeated sprint when athletes slept normally the previous night. Thus, the ergogenic effects of a caffeine dose may depend on the participant’s pre-dose arousal [13, 16, 32]. Contrarily to the former study when N20 enhanced repeated sprint more than CAF after PSD [3], this study favours a moderate dose of caffeine over a short nap when athletes obtained a full-night sleep.

Caffeine ingestion, with or without napping, increased P_min_ compared to N20 and PLA. Despite achieving higher repeated sprint performances, RPE scores were not any higher after CAF and CAF+N20 compared to PLA and N20, indicative of an analgesic effect of caffeine ingestion (*cf.* [20]). Similarly, recent studies [33, 34] reported an enhanced sprint-endurance performance and lower perceived exertion after caffeine mouth-rinsing compared to PLA. Indeed, McLellan et al. [16] reported in their systematic review that up to 80% of the studies showed a positive effect of caffeine on high-intensity efforts lasting longer than 60 seconds. Higher P_min_ during the repeated sprint exercise could be indicative of higher glycolytic contribution in energy synthesis [35]. Actually, all treatments increased post-exercise GLC levels, with higher plasma GLC whenever athletes consumed caffeine, with or without napping. It has been reported that caffeine ingestion induces greater energy availability [3, 21], energy expenditure [36] and oxygen consumption [20] compared to exercise with PLA. However, post-exercise [La] levels were higher only after N20, despite the higher performance after CAF or CAF+N20. This is in line with an earlier study [4] reporting a higher [La] after N20 despite higher performance being achieved after N90. This could indicate higher anaerobic glycolysis after N20 compared to PLA, contrarily to caffeine ingestion that enhanced aerobic glycolysis by way of increased GLC and oxygen availability during CAF and CAF+N20 sessions compared to PLA and N20.

Some participants responded better to CAF than N20, while others performed better after N20 compared to CAF. Interestingly, the combination of caffeine and napping enhanced RAST performance more than each treatment alone, in line with our second hypothesis. Similarly, an earlier study using a similar paradigm reported that CAF+N20 combination produced the highest repeated sprint performance compared to N20 alone and CAF alone [3]. More interestingly, 11 (79%) out of 14 participants have their highest P_min_ during a CAF+N20 session (Fig. 2C). It seems that the short burst of energy after napping combined with the long-term ergogenic effect of caffeine to produce higher performance after the CAF+N20 session compared to CAF alone or N20 alone. Also, it is possible that the caffeine dose mitigated the effect of sleep inertia that may have existed after the nap on performances.

Plasma levels of biomarkers of muscle damage increased whenever participants consumed caffeine with or without napping, confirming our third hypothesis. Post-exercise AST was higher after CAF+N20 compared to N20 and CAF, probably because of the highest physical output during this session. Despite the fact that all interventions enhanced RAST performances, post-exercise URE levels decreased after N20 and increased whenever participants consumed caffeine with or without napping. In fact, URE *per se* is not harmful to muscles; however, its increase reflects greater muscle damage by way of higher muscle protein degradation and/or higher ammoniac (a very toxic waste for muscles). Also, post-exercise CK was higher whenever participants consumed caffeine. Indeed, CK plasma levels depend on the intensity and the duration of the exercise [23]. Higher CK levels could result from the higher sarcolemmal damage subsequent to caffeine ingestion and/or higher power output [23, 37]. From the current results, the higher performance during CAF and CAF+N20 sessions resulted in higher exercise-induced muscle damage. However, an earlier study showed that caffeine consumption increased CK regardless of repeated sprint performance compared to PLA and N20 [3]. It could be that higher muscle damage is a “necessary evil” subsequent to higher performances with caffeine ingestion.

Napping with or without caffeine ingestion enhanced enzymatic antioxidant plasma levels, resulting in better defence against the exercise-induced oxidative stress, which confirms our fourth hypothesis. These results are in line with an earlier study reporting an increase in enzymatic and non-enzymatic antioxidant after napping [7] with or without caffeine ingestion [3]. The exact mechanism behind these findings remains unclear. However, it was suggested that free radicals accumulate during the awake state, and sleep (even for a short episode) promotes their elimination [18]. Also, the current results showed that caffeine ingestion has no prooxidant effects, contrarily to an earlier report using the same dose of caffeine after PSD [3]. It is possible that the same caffeine dose has prooxidant properties after PSD [3] but not after NSN.

### Strengths and limitations

The main strength of the study is that the protocol reflects real-life settings and the study assumptions could be readily used in such conditions. However, the actual sleep duration during the nap opportunity was subjectively rated, which could be a limitation, because sleep onset latency presents variability due to the habitual experience with daytime sleep, prior nocturnal sleep and the time of day. The used paradigm presents a reliable laboratory tool in assessing repeated sprint performance; however, it does not reflect the real sport field settings. Also, no female athletes were recruited in the current study. It has been reported that female athletes respond differently than male to sleep deprivation and rebound sleep [38]. Thus, future studies with more controlled paradigms are warranted to confirm the actual results with more adapted sport-specific tests, larger sample size and on female athletes.

### Practical applications

A great proportion of elite athletes are chronically sleep deprived [39, 40], especially during the time of the COVID-19 pandemic [40–43]. Strategies such as sleep extension and napping showed promising findings in order to mitigate the effects of chronic sleep restriction and/or deprivation [1–4, 7, 38]. From the current study, a moderate dose of caffeine prior to a short post-lunch nap produced a substantial increase in repeated sprint performance after normal sleep, and a similar result was obtained after PSD [3]. However, the exact mechanism behind these findings remains unclear. It is worth noting again that athletes in the current study were caffeine naïve. McLellan et al. [16] reported that the ergogenic effect of the same dose of caffeine could be higher in caffeine naïve participants. Thus, the current results should be treated with caution, and involving regular caffeine consumers may require a higher caffeine dose.

## CONCLUSIONS

A moderate dose of caffeine had greater ergogenic effects on repeated sprint performances than a short post-lunch nap. Probably because of the increased contribution of aerobic glycolysis in energy synthesis, caffeine ingestion resulted in better repeated sprint performance during CAF and CAF+N20 sessions compared to N20 and PLA. Caffeine ingestion resulted in greater muscle damage, and the short nap enhanced antioxidant defence.

## Funding

No external funding related to the project has been received.

## Conflicts of interest/Competing interests

No conflict of interest to disclose.

## Availability of data and material

All data analysed and reported in this study are available from the corresponding author on reasonable request.

## Consent to participate

All participants provided informed consent before participating in the study.

## Consent for publication

All athletes provided consent for anonymous data use for research purposes and publications. All authors approved of the final version to be published and agree to be accountable for any part of the work.
